# Clinical Efficacy of Controlled-Release Morphine Tablets Combined with Celecoxib in Pain Management and the Effects on WNK1 Expression

**DOI:** 10.6061/clinics/2021/e1907

**Published:** 2021-01-11

**Authors:** Jian Li, Fanghai Luan, Jiangfeng Song, Jianhua Dong, Mingfu Shang

**Affiliations:** I Department of Joint Surgery, the Fourth People's Hospital of Jinan, Jinan, China; II Department of Orthopedic Surgery, the Fourth People's Hospital of Jinan, Jinan, China; III Department of Orthopedic, Ju County People's Hospital, Rizhao, China; IV Department of Spinal Cord Repairing, 960 Hospital of the Joint Logistics Support Force of PLA, Jinan, China

**Keywords:** Controlled-release Morphine Tablets, Celecoxib, Osteocarcinoma-Related Pain, Clinical Efficacy, WNK1

## Abstract

**OBJECTIVES:**

This study was designed to evaluate the clinical efficacy of controlled-release morphine tablets combined with celecoxib in relieving osteocarcinoma-related pain and the effects of the combination on WNK1 expression.

**METHODS:**

A total of 110 patients with osteocarcinoma-related pain were selected and divided into two groups based on the treatment administered, including the control group (treated with controlled-release morphine tablets alone) and the study group (treated with a combination of controlled-release morphine tablets and celecoxib). We compared the treatment efficacy, pain level (visual analog scale (VAS)), time of onset of breakthrough pain (BTP), dose of morphine, incidence of adverse events, quality of life (QOL) score, and With-no-lysine 1 (WNK1) expression in the peripheral blood (PB) as determined with qRT-PCR before and after treatment, of the two groups.

**RESULTS:**

The total effective rate of the study group was higher than that of the control group, while the VAS score, time of onset of BTP, dose of morphine, incidence of adverse events, QOL score, and relative WNK1 expression in the PB were lower than those of the control group *(p*<0.05).

**CONCLUSION:**

Combination treatment with controlled-release morphine tablets and celecoxib can be extensively used in the clinical setting because it effectively improves the symptoms, QOL score, and adverse effects in patients with osteocarcinoma-related pain.

## INTRODUCTION

Osteocarcinoma-related pain is unique in that the pain status changes with disease progression. It is one of the most common types of cancer-related pain and affects one-third of all patients with cancer (1,2]. The pain is categorized as rest pain or breakthrough pain (BTP), which severely affects the daily lives of patients and adversely affects their quality of life (QOL) (3). As the molecular mechanism underlying osteosarcoma-related pain is unclear, no treatment strategies have been developed for reducing the adverse events and increasing the tolerance to available drugs, resulting in ineffective pain control in approximately 45% of patients (4). Therefore, to improve the QOL of patients with osteocarcinoma-related pain, it is necessary to determine an effective strategy for controlling pain without severe complications.

Morphine is currently the most common acesodyne, and has been accepted as the gold standard for dealing with cancer-related pain by the European Association for Palliative Care (5). However, the short half-life of morphine necessitates frequent administration. Therefore, efforts are being made to develop controlled-release morphine preparations, which yield a lower daily dose, cause less fluctuations in the serum levels of morphine, reduces the adverse events of morphine, ensure a full night’s sleep, and possibly improve the overall QOL, compared to immediate-release preparations (6). Celecoxib is a non-steroidal anti-inflammatory drug with anti-inflammatory and analgesic effects that are meditated via the suppression of COX-2 (7).

The WNK (With No Lysine kinase) family is so named because it lacks a catalytic lysine that is found in subdomain II of most protein kinases, and the lack of lysine aids in Adenosine triphosphate (ATP) binding (8). The WNK family comprises four members, namely, WNK1, WNK2, WKN3, and WNK4 (9), among which WNK1 is suggested to be closely related to neuropathic pain. It has been identified that the neuropathophysiology is an important factor in osteocarcinoma-related pain (10,11).

Although the analgesic effect of controlled-release morphine tablets combined with celecoxib has been investigated in some studies, few studies have examined the effect of the combination on osteocarcinoma-related pain. In this study, we therefore compared the safety and efficacy of controlled-release morphine tablets combined with celecoxib as a treatment option for osteocarcinoma-related pain, and investigated the effects on WNK1 expression following treatment with controlled-release morphine tablets alone. In this study, we aimed to establish a safer and more effective treatment option for patients with osteocarcinoma-related pain.

## MATERIAL AND METHODS

### Patient selection

A total of 110 patients who received treatment for osteocarcinoma-related pain at our hospital from March 2017 to December 2018 were included as the study subjects. The patients were divided into two groups, namely, the control group and study group, based on the drugs administered. The control group (n=50) was treated with controlled-release morphine tablets alone, and the study group (n=60) received a combination treatment with controlled-release morphine tablets and celecoxib. As per the inclusion criteria, patients with a visual analog scale (VAS) score of ≥7 and aged 50-70 years were included. The exclusion criteria included a history of opioid abuse, communication disorders, contraindications to the study drugs, and current pregnancy/lactation. All the patients and their families agreed to the participation in the study and signed an informed consent. The study was approved by the Ethics Committee of the 960^th^ Hospital of the Joint Logistics Support Force of the PLA.

### Treatment methods

The control group was orally administered controlled-release morphine tablets (Southwestern Pharmaceutical Co., Ltd.; GYZ Zi No. H10930001) at a dose of 10 or 20 mg/day, which was progressively reduced according to the tolerance of the patients. Based on the treatment provided to the control group, celecoxib (Pfizer Pharmaceuticals Ltd.; GYZ Zi No. J20140072) was additionally administered orally, at a dose of 200 mg, twice daily. Both the groups were treated for 21 consecutive days.

### Observation indices

The criteria for evaluating treatment efficacy were as follows: markedly effective was indicated by no pain and clinical symptoms, effective was indicated by the disappearance of pain and improved clinical symptoms, and ineffective was indicated by no obvious improvement in the clinical symptoms and no deterioration in the observed pain and clinical symptoms. The total efficacy rate was calculated by summing the markedly effective and effective percentages.” The total efficacy rate was calculated by summing the markedly effective and effective percentages.

VAS (12) was used to evaluate the intensity of pain in both the groups from 1 day before treatment (T1), and 7 days (T2), 14 days (T3), and 21 days (T4) after treatment (point 0 indicating no pain, points 1-3 indicating mild tolerable pain, points 4-6 indicating pain affecting rest, and points 7-10 indicating intolerable pain affecting sleep and appetite).

The onset times of BTP of both the groups were recorded before and after treatment. The changes in the dose of morphine and the incidence of adverse events were assessed after treatment.

A QOL questionnaire (13) was used for evaluating the QOL at 3 months after discharge based on six items, including gross health (GH), role-physical (RP), physical function (PF), social function (SF), role-emotional (RE), and mental health (MH). The total score for each item was 100, and the total score was found to be positively associated with QOL.

### WNK1 expression in the PB of both groups

A 2.5 mL sample of PB was drawn from the veins in both the groups before and after treatment and stored at −20°C for future analysis. The PB was completely dissolved and the total RNA was extracted with a TRIzol kit (CD-13433-ML; Wuhan Chundu Biotechnology Co., Ltd., China) according to the manufacturer’s instructions.

The concentration and purity of the total mRNA were determined using a DR5000 ultraviolet visible spectrophotometer and reverse transcribed with a reverse transcription kit (KR123, Tiangen Biotech (Beijing) Co., Ltd., China) in strict accordance with the manufacturer’s instructions. PCR was performed using β-actin as the internal reference. The PCR comprised 2×FastKing One Step RT-PCR MasterMix (25 μL), 25× RT-PCR Enzyme Mix (2 μL), 5′-primer (1.25 μL), 3′-primer (10 ng/μL), and water, up to a final volume of 50 μL. The conditions of PCR were as follows: 40 cycles of pre-denaturation at 95°C for 3 min, 94°C for 30 s, 60°C for 30 s, and 72°C for 30 s. The data were analyzed using the 2^−△△ct^ method (14). The primers for WNK1 and β-actin were designed and synthetized by Takara Biotechnology Co. Ltd. (China), and their sequences are enlisted in Table 1.

### Statistical analyses

For the experimental data, the statistical analyses were performed with SPSS version 21.0 (IBM Corp, Armonk, NY, USA), and GraphPad Prism7 was used for drawing the plots. The nominal data were analyzed using the chi-square test, and the data are presented as the mean±standard deviation (x±SD). Multiple groups were compared using independent samples *t*-test, while comparisons between two groups were compared using Dunnett’s test. For all the statistical comparisons, *p*<0.05 was considered to be significant.

## RESULTS

### Comparison of general characteristics

There were no differences between the groups with respect to the general characteristics, including sex, age, body weight, disease course, educational background, dietary preference, domicile, exercise habits, history of marriage, smoking, and alcohol consumption (*p*>0.05, Table 2).

### Comparison of treatment efficacy

The treatment was found to be markedly effective in 36 patients (60.00%), effective in 20 (33.33%) patients, and ineffective in 4 (6.67%) patients in the study group, resulting in a total effective rate of 93.33%, which was higher than that of the control group. In the control group, the total effective rate was 78.00%, being markedly effective in 23 (46.00%) patients, effective in 16 (32.00%) patients, and ineffective in 11 (22.00%) patients; *p*<0.05, Table 3].

### Comparison of VAS scores at different time points

The VAS scores of the two groups significantly reduced at T2, T3, and T4 (*p*<0.05) compared with those at T1, and there were no obvious intergroup differences (*p*>0.05). The VAS scores at T3 and T4 were significantly lower than those at T2 (*p*<0.05), while the VAS scores at T4 were significantly lower than those at T3 (*p*<0.05). The VAS scores of the study group were lower than those of the control group at T2, T3, and T4 (*p*<0.05, Table 4).

### Comparison of time of onset of BTP at different time points

The time of onset of BTP in the two groups was significantly reduced at T2, T3, and T4 (*p*<0.05) compared with that at T1, and there were no obvious intergroup differences (*p*>0.05). The time of onset of BTP at T3 and T4 was significantly lower than that at T2 (*p*<0.05), while the onset time of BTP at T4 was significantly lower than that at T3 (*p*<0.05). The onset time of BTP in the study group was lower than that of the control group at T2, T3, and T4 (*p*<0.05, Table 5).

### Comparison of the dose of morphine at different time points

The dose of morphine of the two groups was significantly reduced at T3 and T4 (*p*<0.05) compared to that at T2, and the dose of morphine at T4 was significantly lower than that at T3 *(p*<0.05). The study group required a lower dose of morphine than the control group at T2, T3, and T4 (*p*<0.05, Table 6).

### Comparison of the incidence of adverse events

There were no significant differences between the groups with respect to the incidence of adverse events following treatment, including nausea, vomiting, constipation, respiratory depression, and edema (*p*>0.05). There were fewer cases of drowsiness, dizziness, and dysuresia in the study group than in the control group (*p*<0.05, Table 7).

### Comparison of post-treatment QOL

**Figure 1:**
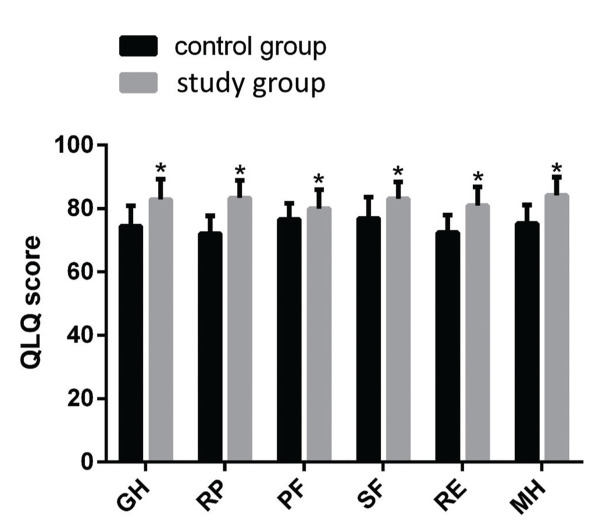
Comparison of the QOL of the two groups post-treatment. Note: *p<0.05 compared with the control group. GH: gross health; RP: role-physical; PF: physical function; SF: social function; RE: role-emotional; and MH: mental health.

After treatment, the GH, RP, PF, SF, RE, and MH scores were 82.84±6.45, 83.34±5.56, 83.45±5.88, 83.13±5.34, 80.92±5.98, and 84.21±5.78, respectively, in the study group and 72.13±5.56, 76.61±5.13, 76.93±6.67, 72.42±5.54, and 75.33±5.78, respectively, in the control group (*p*<0.05, Fig. 1).

### Intergroup comparison of relative WNK1 expression in PB

**Figure 2:**
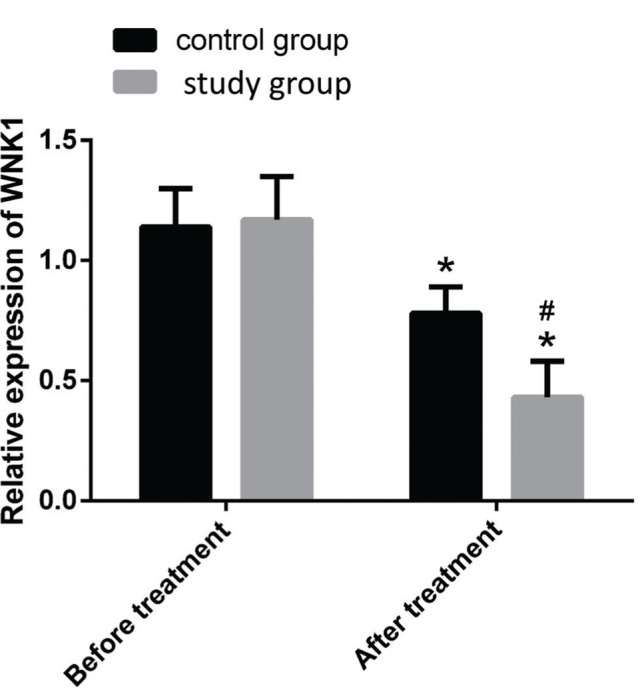
Comparison of the relative expression of WNK1 in the PB of the two groups before and after treatment. Note: *p<0.05 for intragroup comparison with conditions before treatment, and *^#^p<*0.05 compared with the control group.

The relative expression of WNK1 in the PB of the control and study groups was 1.14±0.16 and 1.17±0.18, respectively, before treatment (*p*>0.05), and 0.78±0.11 and 0.43±0.15, respectively, after treatment. The relative expression of WNK1 in the study group was significantly lower than that of the control group after treatment (*p*<0.05, Fig. 2).

## DISCUSSION

Despite the severe impact of osteocarcinoma-related pain on the QOL of patients, and the heavy burden on the patients and the healthcare system, the underlying molecular mechanism remains to be elucidated (15). Osteosarcoma-related pain is primarily treated with opioids; however, their effects are compromised by the incidence of adverse events, including respiratory depression, poor tolerance, dependence, and habituation, which limits their use in pain relief (16). Therefore, the identification of other therapeutic strategies for controlling osteocarcinoma-related pain is crucial for improving the QOL of patients with this type of pain.

Synergistic, additional, or antagonistic effects are observed when two analgesics are administered simultaneously. In the first case, an equivalent or greater analgesic effect can be achieved when the doses of the drugs are low (17). In a previous study, 342 patients with metastatic osteocarcinoma-related pain were divided into three groups and treated with controlled-release diclofenac+celecoxib+morphine tablets, controlled-release diclofenac+morphine tablets, and controlled-release celecoxib+morphine tablets, for analyzing their clinical effects. The results demonstrated that the controlled-release diclofenac+celecoxib+morphine tablets significantly reduced the VAS score, time of onset of BTP, dose of morphine, and the incidence of adverse events while increasing the rate of odynolysis, compared to the other combinations (18). The results of this study demonstrated that the total effective rate of treatment of the study group was higher than that of the control group, while the VAS score and time of onset of BTP of the study group after treatment were lower than those of the control group. These results indicated that the combination of controlled-release morphine tablets and celecoxib can effectively control osteocarcinoma-related pain. The dose of morphine and the incidence of adverse events of the two groups were subsequently compared, and the results demonstrated that the morphine requirement of the study group was lower than that of the control group. Additionally, fewer adverse events, like drowsiness, dizziness, and dysuresia were observed in the study group. Further investigation of the underlying causes revealed that osteocarcinoma-related pain is a result of multiple factors. The two drugs considered in this study have different analgesic mechanisms. The anti-inflammatory and analgesic effects of celecoxib are mediated via the reduction of prostaglandin levels resulting from the inhibition of COX-2 activity (19). However, morphine suppresses the release of excitatory transmitters and reduces the degree of analgesia, which causes the opioid receptors in the spinal cord, ventricle, and thalamus to bind to each other for producing analgesia (20). The analgesic mechanisms of the two drugs are independent of each other and they act simultaneously for effectively controlling pain and reducing the adverse events related to dose-reduced morphine administration.

WNK1 is a newly discovered ion channel regulatory protein, and its effect on hypertension is being investigated (21-23). However, recent reports indicate that WNK plays a role in pain generation, and some studies have demonstrated that INKCC1/WNK1/WNK1HSN2 is involved in the development of neuropathic pain caused by spinal injury and can be regulated for therapeutic purposes (24). More recent studies have revealed that the expression of WNK1 is upregulated in the spinal cord and neurons of the dorsal root ganglion in rats with osteocarcinoma-related pain. It has been demonstrated in a rat model that the intrathecal injection of WNK1 siRNA or closantel (WNK1-SPAK or OSR1 inhibitor) improves pain behavior. Based on this observation, researchers have estimated that the inhibition of WNK1/SPAK/OSR1 can potentially alleviate osteocarcinoma-related pain (25). The results of this study demonstrated that the relative expression of WNK1 in the PB was significantly reduced in both the groups following treatment, and the reduction was more significant in the study group. This indicated that combination treatment with controlled-release morphine tablets and celecoxib can reduce osteosarcoma-related pain.

The patients in the study group were selected in strict accordance with the inclusion and exclusion criteria, and there was no significant differences in the general characteristics of the two groups. This eliminated the possibility of deviations in the experimental results arising from differences in the characteristics of the study subjects in the two groups. The results of this study demonstrated the safety and efficacy of controlled-release morphine tablets and celecoxib as a combinatorial treatment for osteocarcinoma-related pain. However, this study has certain limitations, including the fact that the optimal dose for the treatment of osteocarcinoma-related pain has not been discussed and the study does not elucidate the associations among osteocarcinoma-related pain, WNK1 expression, efficacy of controlled-release morphine tablets, and the efficacy of celecoxib, which should be investigated in future studies.

In conclusion, the results of our study demonstrated that controlled-release morphine tablets and celecoxib is an effective combination therapy for clinical use as it reduces the degree of osteocarcinoma-related pain and adverse events, and improves the QOL of the patients. However, the relatively small sample size considered herein may have introduced a bias in the results, and more studies are necessary in the future for validating the results obtained herein.

## AUTHOR CONTRIBUTIONS

Li J, Luan F, and Song J contributed in concept and design of the manuscript. Song J and Dong J contributed in data collection. Song J and Dong J contributed in data analysis and interpretation. Li J and Luan F contributed in article drafting. Shang M contributed in critical revision of article. Approval of article: All authors.

## Figures and Tables

**Table 1:** Primer sequences.

Gene	Upstream	Downstream
*WNK1*	5′-CAGAGTGAG-CAGCCAACAGA-3′	5′-CCACGGACTGAG-GCATACTT-3′
*β-Actin*	5′-CACCCGCGAGTACAACCTTC-3′	5′-CCCATACCCACCATCACACC-3′

**Table 2:** Comparison of the general characteristics of the two groups ([n(%)], x ± SD).

Characteristics	Control group (n=50)	Study group (n=60)	χ^2^/F	*p*
Sex			0.642	0.423
Male	28 (56.00)	29 (48.33)		
Female	22 (44.00)	31 (51.67)		
Age (years)	59.24±7.67	61.45±8.11	1.458	0.148
Weight (kg)	62.56±6.67	64.66±6.21	1.707	0.091
Disease course (y)	1.67±0.68	1.54±0.71	0.975	0.332
Educational background			1.243	0.265
<Senior middle school	23 (46.00)	34 (56.67)		
≥ Junior high school	27 (54.00)	26 (43.33)		
Dietary preference			1.430	0.232
Light	37 (74.00)	38 (63.33)		
High fat	13 (26.00)	22 (36.67)		
Domicile			0.376	0.540
Urban	34 (68.00)	44 (73.33)		
Rural	16 (32.00)	34 (26.67)		
Habit of exercising			0.362	0.547
Yes	22 (44.00)	23 (38.33)		
No	28 (56.00)	37 (61.67)		
Marital status			0.069	0.966
Married	44 (88.00)	52 (86.67)		
Unmarried	2 (4.00)	3 (5.00)		
Divorced	4 (8.00)	5 (8.33)		
History of smoking			0.543	0.461
Yes	35 (70.00)	38 (63.33)		
No	15 (30.00)	22 (36.67)		
History of alcohol consumption			0.240	0.624
Yes	29 (58.00)	32 (53.33)		
No	21 (42.00)	28 (46.67)		

**Table 3:** Comparison of the treatment efficacy of the two groups.

Group	Markedly effective	Effective	Ineffective	Total effective rate
Control group (n=50)	23 (46.00)	16 (32.00)	11 (22.00)	78.00%
Study group (n=60)	36 (60.00)	20 (33.33)	4 (6.67)	93.33%
χ^2^	-	-	-	5.445
*p*	-	-	-	0.020

**Table 4:** Comparison of the visual analog scale (VAS) score of the two groups at different time points (point, x ± SD).

	Time points			
Group	T1	T2	T3	T4
Control group (n=50)	8.67±0.78	5.65±0.78^a^	4.56±0.63^a^ ^b^	3.45±0.73^a^ ^b^ ^c^
Study group (n=78)	8.44±0.89	4.21±0.67^a^	3.44±0.59^a^ ^b^	2.67±0.56^a^ ^b^ ^c^
T	1.427	10.416	9.613	6.338
*p*	0.157	<0.001	<0.001	<0.001

a Note: *p*<0.05 for intragroup comparison at T1;

b *p*<0.05 for intragroup comparison at T2;

c *p<*0.05 for intragroup comparison at T3.

**Table 5:** Comparison of the time of onset of BTP of the two groups at different time points (times, x ± SD).

	Time points			
Group	T1	T2	T3	T4
Control group (n=50)	4.13±0.80	3.57±0.82^a^	3.25±0.74^a^ ^b^	2.72±0.89^a^ ^b^ ^c^
Study group (n=78)	4.35±0.71	2.85±0.78^a^	2.41±0.71^a^ ^b^	1.81±0.78^a^ ^b^ ^c^
T	1.528	4.710	6.061	5.714
*p*	0.130	<0.001	<0.001	<0.001

a Note: *p*<0.05 for intragroup comparison at T1;

b *p*<0.05 for intragroup comparison at T2;

c *p*<0.05 for intragroup comparison at T3.

**Table 6:** Comparison of the dose of morphine of the two groups at different time points after treatment (mg, x ± SD).

	Time points			
Group	T2	T3	T4
Control group (n=50)	83.34±17.56	104.53±16.24^a^	142.54±23.72^a^ ^b^
Study group (n=78)	54.21±16.05	75.67±18.12^a^	101.52±21.56^a^ ^b^
T	9.081	8.716	9.493
*p*	<0.001	<0.001	<0.001

a Note: *p*<0.05 for intragroup comparison at T2;

b *p*<0.05 for intragroup comparison at T3.

**Table 7:** Comparison of the incidence of adverse events in the two groups after treatment [n(%)].

Adverse events	Control group(n=50)	Study group(n=78)	χ^2^	*p*
Nausea and vomiting	7 (14.00)	7 (11.67)	0.134	0.715
Drowsiness	12 (24.00)	5 (8.33)	5.123	0.024
Dizziness	16 (32.00)	7 (11.67)	6.818	0.009
Constipation	3 (6.00)	6 (10.00)	0.581	0.446
Respiratory depression	2 (4.00)	1 (1.67)	0.560	0.454
Edema	3 (6.00)	2 (3.33)	0.447	0.504
Dysuresia	7 (14.00)	2 (3.33)	4.131	0.042
